# Dysregulated Calcium Handling in Cirrhotic Cardiomyopathy

**DOI:** 10.3390/biomedicines11071895

**Published:** 2023-07-04

**Authors:** Sang Youn Hwang, Hongqun Liu, Samuel S. Lee

**Affiliations:** 1 Liver Unit, University of Calgary Cumming School of Medicine, Calgary, AB T2N 4N1, Canada; mongmani@daum.net (S.Y.H.); hliu@ucalgary.ca (H.L.); 2 Department of Internal Medicine, Dongnam Institute of Radiological & Medical Sciences, Busan 46033, Republic of Korea

**Keywords:** cirrhotic cardiomyopathy, L-type calcium channel, ryanodine receptors, cardiac contractility, calcium transient

## Abstract

Cirrhotic cardiomyopathy is a syndrome of blunted cardiac systolic and diastolic function in patients with cirrhosis. However, the mechanisms remain incompletely known. Since contractility and relaxation depend on cardiomyocyte calcium transients, any factors that impact cardiac contractile and relaxation functions act eventually through calcium transients. In addition, calcium transients play an important role in cardiac arrhythmias. The present review summarizes the calcium handling system and its role in cardiac function in cirrhotic cardiomyopathy and its mechanisms. The calcium handling system includes calcium channels on the sarcolemmal plasma membrane of cardiomyocytes, the intracellular calcium-regulatory apparatus, and pertinent proteins in the cytosol. L-type calcium channels, the main calcium channel in the plasma membrane of cardiomyocytes, are decreased in the cirrhotic heart, and the calcium current is decreased during the action potential both at baseline and under stimulation of beta-adrenergic receptors, which reduces the signal to calcium-induced calcium release. The study of sarcomere length fluctuations and calcium transients demonstrated that calcium leakage exists in cirrhotic cardiomyocytes, which decreases the amount of calcium storage in the sarcoplasmic reticulum (SR). The decreased storage of calcium in the SR underlies the reduced calcium released from the SR, which results in decreased cardiac contractility. Based on studies of heart failure with non-cirrhotic cardiomyopathy, it is believed that the calcium leakage is due to the destabilization of interdomain interactions (dispersion) of ryanodine receptors (RyRs). A similar dispersion of RyRs may also play an important role in reduced contractility. Multiple defects in calcium handling thus contribute to the pathogenesis of cirrhotic cardiomyopathy.

## 1. Introduction

Cardiovascular abnormalities in cirrhosis include hyperdynamic circulation and cirrhotic cardiomyopathy (CCM); the former is the combination of decreased peripheral vascular resistance and increased cardiac output whereas the latter is a syndrome of cardiac contractile dysfunction in the absence of a primary cardiac condition [1,2,3]. There are two sets of diagnostic criteria for CCM: the World Congress of Gastroenterology (WCG, 2005) criteria and the Cirrhotic Cardiomyopathy Consortium (CCC, 2019) criteria. The latter is based on advanced imaging methods. Whether the new criteria are superior to the old is not yet settled, although the current consensus seems to favor the new criteria. Table 1 lists the comparisons between the two CCM diagnostic criteria.

The pathophysiology of the cardiovascular system is complex. In cirrhotic patients and animal models, the abnormal architecture of the liver causes portal venous hypertension, and the congested gut results in increased intestinal permeability. These pathophysiological changes trigger bacterial overgrowth, translocation, and increased bacteria-derived products in their circulation, including endotoxins that elevate systemic pro-inflammatory cytokines, such as tumor necrosis factor alpha (TNFα), interleukin-1β (IL-1β) and interleukin-6 (IL-6). Thus, patients with cirrhosis and portal hypertension manifest an inflammatory phenotype that leads to increased vasodilators such as nitric oxide, carbon monoxide, prostaglandins, bile acids, and glucagon. Therefore, the peripheral vasculature is dilated.

It is well noted that the sympathetic nervous system (SNS) tone is increased in cirrhotic patients (serum concentration of norepinephrine is increased) [11]. The increased activity of the SNS and elevated serum concentrations of vasoconstrictive catecholamines are a compensatory reaction to vasodilatation, not its cause. In cirrhosis, vasodilators predominate over vasoconstrictors, and, therefore, the phenotype of cirrhotic patients and animal models is systemic vasodilatation. Because of the baseline peripheral vasodilatation of cirrhotic patients, cardiac dysfunction is subclinical at rest, which results in delayed or missed diagnosis in clinically stable patients with cirrhosis. However, when patients face cardiovascular challenges such as liver transplantation, transjugular intrahepatic portosystemic shunt (TIPS) insertion, hemorrhage, and vasoactive drugs [1], cardiac dysfunction manifests overtly.

There are numerous pathogenic mechanisms underlying CCM which broadly comprise two distinct pathways: factors related to (a) liver dysfunction with synthetic failure/dysregulation of substances such as proteins, lipids, lectins, hormones, etc., and (b) portal hypertension with gut congestion and induction of the inflammatory phenotype. A detailed discussion of these mechanisms is beyond the scope of this current review on calcium dysregulation (the interested reader is referred to our recent review on the mechanisms of CCM [12], however, some important mechanisms are briefly summarized below.

Uhlig and coworkers [13] demonstrated that serum TNFα and cardiac inflammatory cells are significantly increased in cirrhotic rats. Our lab [14] showed that Fas protein expression and PARP cleavage, which are indices of apoptosis, are significantly increased in the cirrhotic hearts of rats. Moreover, anti-FasL monoclonal antibody injection in BDL-mice improved systolic and diastolic dysfunction. Thus, the study implies that apoptosis plays an important pathogenic role in murine cardiac dysfunction. Another study [15] on oxidative stress revealed that oxidative stress-related parameters are increased and antioxidant regulation is decreased in cirrhotic hearts. Erythropoietin, an antioxidant, significantly downregulated oxidative stress and reversed impaired cardiac function. Structural changes in cirrhotic rat hearts included alterations in myosin heavy chain (MHC) isoforms, leading to a dominance of weaker-contracting β-MHC over the more powerful α-MHC [16] and increased cardiac fibrosis [13].

Among the ions involved in cardiac dysfunction in cirrhosis, calcium transients play an essential role. The present review summarizes the dysregulated calcium handling in cirrhotic cardiomyocytes.

## 2. Calcium Transport in Cardiac Excitation–Contraction Coupling

**Figure 1:**
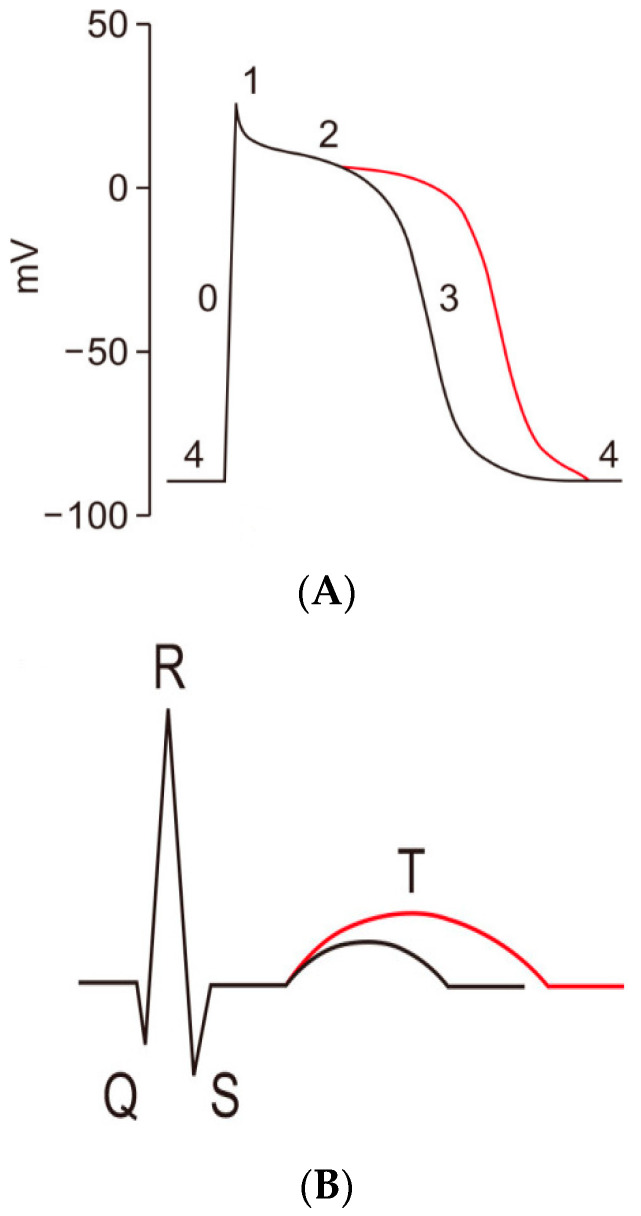
Action potential of ventricular myocardium (**A**) and corresponding electrocardiogram (ECG) (**B**). Example of a normal ventricular myocardial action potential with normal QT interval (black line) and a prolonged ventricular action potential due to an I_Kr_ block resulting in a prolonged phase 2 and 3 of the action potential and prolonged QT interval (red line). Phase 0: rapid depolarization mediated by voltage-gated sodium channels (I_Na_) corresponding with the start of the QRS complex on the ECG. Phase 1: closure of the sodium channels. Short transient outward current (I_to_) mediated by voltage-gated potassium channels. Phase 2: plateau phase due to an equilibrium between the inward calcium current by L-type calcium channels and an outward potassium current (I_Kur_ and I_Ks_). Phase 3: repolarization due to closure of the L-type calcium channels and outward potassium currents (I_Ks_ and I_Kr_) corresponding to the end of the T-wave on the ECG. Phase 4: a slow depolarization occurs if the current reaches the threshold, which initiates the next depolarization (Reproduced from Lee W. et al. [19]).

The cardiac action potential is a brief change in voltage across the plasma membrane of cardiac myocytes. In a normal heart, the action potential of the ventricular myocardium triggers cardiac contraction. There are five phases in the action potential of the ventricular myocardium (Figure 1). Our previous studies have demonstrated abnormalities of two ion transients, calcium [16,17] and potassium [18], in rat cirrhotic ventricular myocytes.

**Figure 2:**
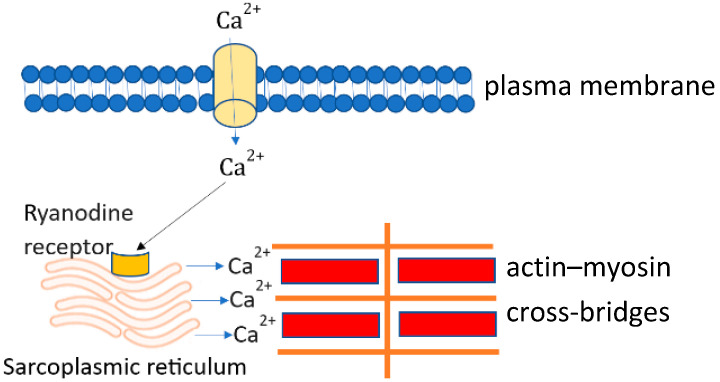
Calcium transients in cardiomyocytes.

Calcium entering the cytoplasm via L-type calcium channels triggers the opening of calcium release channels (ryanodine receptor), leading to calcium release from the sarcoplasmic reticulum (SR). The released calcium binds troponin C, which enables the movement of tropomyosin and thus exposes the myosin binding site on the thin filaments (Figure 2) [20]. A cross-bridge is then formed between the thick and thin filaments which results in cardiac contraction [21]. After contraction, both voltage-gated calcium channels and calcium release channels are closed, and the calcium is removed from the cytosol via the sarcoplasmic-endoplasmic reticulum calcium-ATPase (SERCA) to the SR and via the sodium–calcium exchanger (NCX) outside of the cardiomyocyte [22]. In the steady state, the amount of calcium entering the cell must be equal to that extruded in each cardiac cycle. If not, the cell would either gain or lose calcium [23]. Calcium transport is abnormal in cirrhotic cardiomyopathy [16,17].

At phase 2 of the action potential, calcium enters the cytosol through L-type calcium channels which activate ryanodine receptors (calcium-induced calcium release channels). Activation of ryanodine receptors triggers the release of calcium from the sarcoplasmic reticulum. Calcium released mainly from the sarcoplasmic reticulum combines with the troponin complex and triggers actin–myosin cross-bridge linking, and thus cell contraction. This process is called excitation–contraction coupling.

## 3. Abnormal Membrane L-Type Calcium Channels in Cirrhotic Cardiomyopathy

Calcium is an essential second messenger in all living organisms. From Caenorhabditis elegans to mammals, calcium is mandatory for body functions, locomotion, and neural activities [24]. In the late nineteenth century, Sidney Ringer published his observation which demonstrated that calcium is essential for cardiac contraction: the removal of calcium from the perfusion buffer of the frog heart halted cardiac contraction [25]. The force of cardiac contractility is calcium concentration-dependent [16]. This experiment implied that external calcium is required for cardiac systole. L-type calcium channels are the channels through which calcium outside the cell enters the cytosol. L-type calcium channels are essential for the initiation and regulation of excitation–contraction (EC) coupling in cardiomyocytes. The rapid entry of calcium via these channels triggers the release of intracellular calcium from ryanodine receptors embedded on the surface of the SR. The released calcium activates the myofilaments and finally triggers the contraction of cardiomyocytes [20]. Any abnormality of L-type calcium channels impacts the amount of cytosolic calcium prior to the contraction and therefore affects contractility.

**Figure 3:**
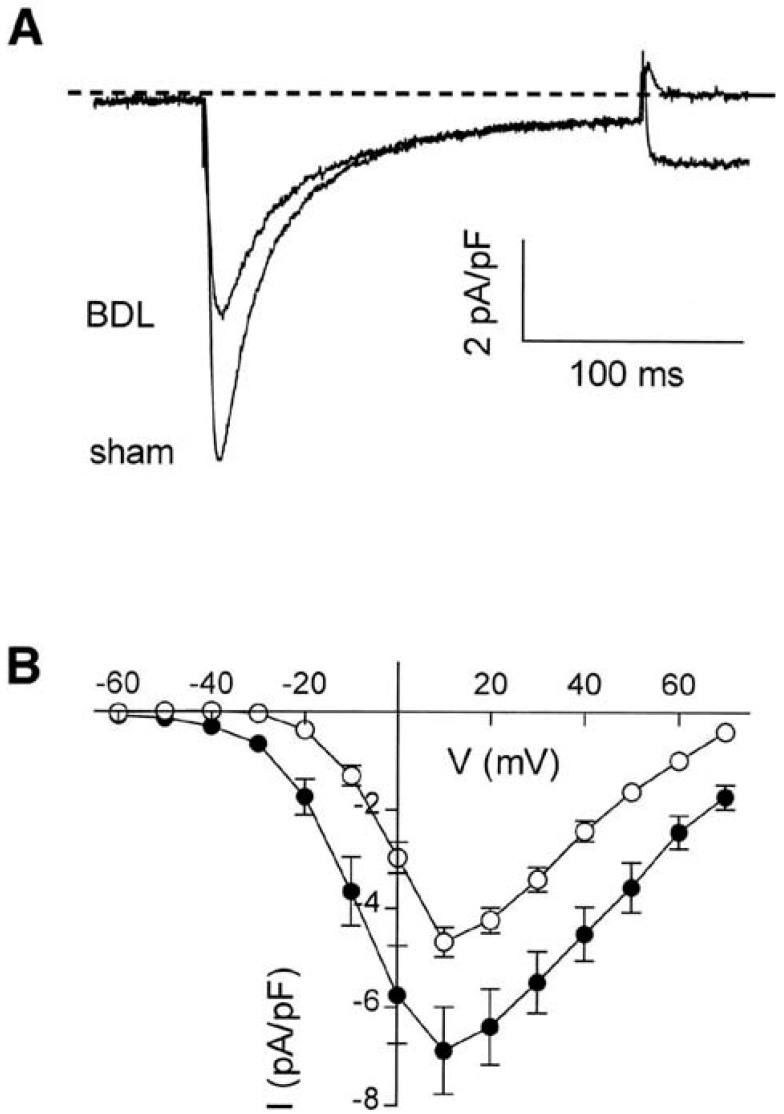
Calcium kinetics in BDL-cirrhotic and control cardiomyocytes. Comparison of L-type Ca^2+^ current densities from ventricular myocytes of BDL- and sham-treated animals. (**A**) Representative currents elicited by a depolarizing step to +10 mV from a conditioning potential of −40 mV. All currents were corrected for cell capacitance and expressed as current densities. (**B**) Current–voltage relationships were constructed by depolarizing steps to test potentials between −60 and +70 mV from the conditioning potential of −40 mV for myocytes isolated from BDL-treated (*n* = 19; O) and sham-operated (*n* = 16; ●) animals (Reproduced from Ward CA et al. [17]).

The abundance and current of L-type calcium channels are decreased following the development of cardiac dysfunction. Mukherjee et al. [26] studied pacing-induced chronic heart failure in pigs. They reported that two weeks after pacing (240 bpm), the content of L-type calcium channels was significantly decreased compared to controls (Bmax. 149 ± 16 vs. 180 ± 12 fmol/mg, *p* < 0.03) as was the current of L-type calcium channels (2.47 ± 0.10 vs. 3.63 ± 0.25 pA/pF, *p* < 0.02). Our study [17] on cirrhotic cardiomyopathy in rats showed a similar pattern to the Mukherjee study. In Sprague Dawley rats, a model of cirrhotic cardiomyopathy was created by bile duct ligation (BDL). We demonstrated that the protein expression of L-type calcium channels in cirrhotic hearts is significantly reduced compared with controls, and the peak inward calcium current was significantly reduced (−4.9 ± 0.3 pA/pF vs. −7.3 ± 0.9 pA/pF, *p* < 0.0001) [17]. At all membrane potentials examined, the current densities of calcium influx entering via L-type calcium channels were consistently lower in cardiomyocytes measured from cirrhotic animals than that from sham controls (Figure 3).

The response of peak inward calcium current to maximal isoproterenol stimulation was also significantly lower. Protein expression and messenger RNA transcription for RyR2, SERCA2, and calsequestrin were quantitatively unchanged [17].

## 4. Abnormal Intracellular Calcium Handling System

It is now clear that the density and function of L-type calcium channels are reduced in cardiomyocytes from cirrhotic rats. This abnormality impacts the calcium transients in the intracellular calcium handling system, mainly ryanodine receptors (RyRs). RyR is a homotetrameric intracellular calcium release channel that is composed of four 565-kDa protomers and is located on the SR in cardiomyocytes and other cell types [24]. The name derives from its ability to bind ryanodine, a component of the *Ryania speciosa* plant used by South American Indigenous people in blow darts to paralyze prey. When ryanodine binds RyR channels, they induce an open state which results in an uncontrolled release of calcium from the SR, leading to muscle tetany. The flux of cytosolic calcium transients, calcium influx, and efflux should be balanced to maintain normal contractile function. RyR dysfunction is associated with a variety of conditions such as cardiac arrhythmias and heart failure. Although there are no structural changes in the intracellular calcium handling system in cirrhotic cardiomyopathy [17], the intracellular calcium handling system is disorganized by phenomena such as dispersion.

The functional unit (calcium release unit) is composed of the L-type calcium channel and ryanodine receptor clusters (RyR clusters) which play an essential role in regulating calcium transients. In an in vivo study, Kolstad and co-workers [27] showed that RyR clusters are abnormal in structure and function in cardiomyocytes from heart failure. They isolated and tested the RyR status in cardiomyocytes from rats with infarction-derived heart failure. Using super-resolution imaging, they observed that RyR clusters in failing cardiomyocytes showed dispersion, which resulted in more numerous, smaller clusters. Compared with sham controls, RyR clusters from heart failure produced weaker calcium sparks, calcium uptake was decreased, releasable SR calcium content was reduced, and leakage was increased. All these changes resulted in slow calcium kinetics leading to a weakened cardiac contraction in heart failure. This group further tested the role of the over-stimulation of beta-adrenergic receptors in the dispersion of RyRs [28]. They used isoproterenol to stimulate ventricular cardiomyocytes isolated from rats. They showed that long time stimulation with isoproterenol caused the dispersion of RyR clusters in the SR. The dispersion of RyR reduced calcium spark fidelity and magnitude. Furthermore, the dispersion of RyR also increased the ‘silent’ calcium leak. These changes resulted in smaller and desynchronized calcium transients, which are hallmarks of cardiac dysfunction.

It is well known that the sympathetic nervous system is over-activated in patients with cirrhosis. In the early 1960s, Shaldon et al. [29] measured the level of catecholamines, an index of sympathetic nervous activity, and showed that this is increased in portal venous plasma. Many other studies confirmed that the sympathetic nervous system is overactivated in patients with cirrhosis [30]. The overactivated sympathetic nervous system stimulates beta-adrenergic receptors which may cause dispersion of RyR, thus leading to contractile dysfunction in the cirrhotic heart.

The ventricular systolic force depends on the magnitude of the cytosolic calcium transient and the sensitivity of the contractile proteins to the calcium. We showed that cellular calcium transients are reduced in cardiomyocytes from BDL-cirrhotic rats, which explains the decreased systolic force in these animals [16]. Another factor in the decreased sensitivity of myofilament to calcium in cirrhotic cardiomyocytes is the shift from an alpha-myosin heavy chain (α-MHC) to β-MHC. The α-MHC isoform contracts more vigorously than the β-MHC, albeit at the cost of utilizing more energy. Furthermore, we showed that calcium sensitivity was more attenuated in β-MHC than in α-MHC containing fibers [31]. Our study revealed that the α-MHC isoform predominates in myosin heavy chain in sham-control rat hearts (90%), whereas, in comparison, the predominant phenotype is β-MHC in the BDL-cirrhotic heart [16]. This suggests that the shift from α-MHC to β-MHC in the cirrhotic heart plays an important role in the suppressed cardiac contractility of the cirrhotic heart [32].

Titin, a giant muscle protein, is mainly involved in ventricular diastolic function. In effect, its ‘spring-like’ elasticity is responsible for much of the passive mechanical recoil of early diastole. Kellermayer et al. demonstrated that titin abnormalities are associated with dilated cardiomyopathy. Our group did not find any direct titin structural abnormalities in cirrhotic rat hearts. However, we showed that a titin modulator, protein kinase A, is decreased, which may contribute to abnormal titin function and thus reduced diastolic compliance in the cirrhotic ventricle [33].

**Figure 4:**
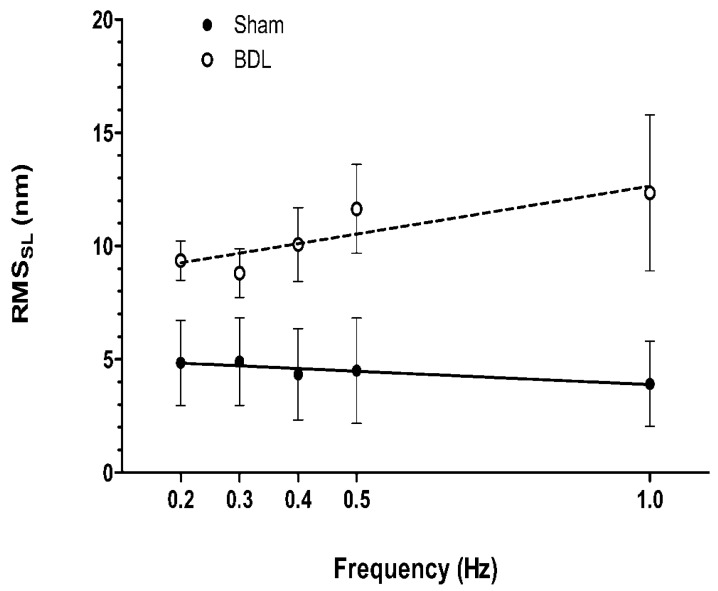
Spontaneous sarcomere activity in sham-control and BDL-cirrhotic rat ventricular trabeculae and spontaneous sarcomere fluctuation at 26 °C during the end-diastolic period in trabeculae at stimulus rates from 0.2 to 1 Hz. Open symbols are the mean ± SEM of RMS_SL_ and lines represent the linear relation between the frequency of stimulation and the amount of spontaneous sarcomere activity. [Ca^2+^]o was 0.5 mmol/L; *n* = 5. BDL-cirrhotic trabeculae show significantly greater spontaneous sarcomere fluctuation, suggesting increased calcium leakage (Reproduced from Honar H. et al. [16]).

Another abnormality of the intracellular calcium handling system is the calcium leakage from the SR. Calcium leakage reduces cardiac contractility in heart failure [34]. We examined the root mean square value of sarcomere length fluctuations (RMS_SL_) to evaluate the amount of spontaneous sarcomere length fluctuation during diastole and found that RMS_SL_ is significantly higher in ventricular trabeculae from cirrhotic rat hearts at all stimulus rates compared with that from sham-control rats [16] (Figure 4). This implies that the leakage of calcium from the SR in cirrhotic cardiomyocytes is higher than that from sham controls. Following contraction, the calcium leaks during diastole. In normal conditions, the random opening of RyR gives rise to calcium sparks and the calcium wave is small. However, in conditions such as congestive heart failure, the probability of spontaneous diastolic opening of SR calcium channels is increased, which causes noticeable fluctuations of diastolic sarcomere length and also the propagation of contractile waves in the myocytes [35]. Our study demonstrated the increased spontaneous fluctuations of sarcomere length during diastole in BDL trabeculae, which suggests that there are dispersions of RyR [16]. In addition, Obayashi et al. [34] reported that the decreased force of cardiac contractility is proportional to the amount of spontaneous activity in chronic heart failure in rats. Our data indicate that spontaneous SR-calcium release contributes to the reduction in inotropism in cirrhotic hearts. In addition, spontaneous random diastolic calcium release hinders the synchronization of individual sarcomeres, which also decreases the contractile force of cirrhotic hearts.

In addition to cardiac contractile dysfunction, calcium leakage also results in arrhythmias [24]. Calcium leakage causes delayed after-depolarizations (DADs) of the cell membrane due to the activity of the sodium–calcium exchanger. One such arrhythmia is atrial fibrillation [36]. It has been documented that atrial fibrillation is the most common arrhythmia found in patients with cirrhosis, with a prevalence between 6.6% and 14.2% [37,38]. Furthermore, atrial fibrillation is the most common arrhythmia in the perioperative period of both cirrhotic and noncirrhotic patients undergoing major surgery such as liver transplantation [39]. Atrial fibrillation is related to a variety of adverse outcomes [40] such as stroke, acute kidney injury, and in-hospital mortality. Understanding the role of calcium leakage in atrial fibrillation may eventually lead to new strategies to treat/prevent this arrhythmia in patients with cirrhosis.

## 5. Abnormal Calcium Handling Prolongs Cardiomyocyte Contraction/Relaxation

There are several theories to explain the mechanism of prolonged contraction/relaxation in the failing heart, such as the prolongation of the action potential duration [41] and changes in myofibrillar proteins [16].

**Figure 5:**
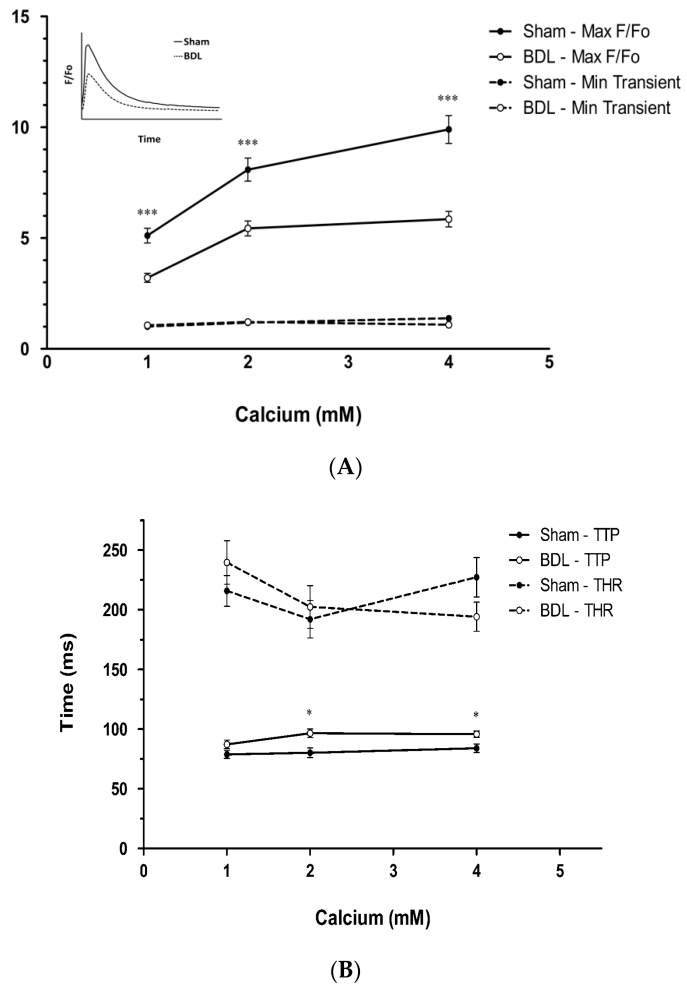
Force generation and calcium kinetics in isolated ventricular trabeculae from BDL-cirrhotic and control rat hearts. (**A**): Ca^2+^ transient fluorescence in isolated myocytes during steady-state contraction at a stimulus rate of 0.5 Hz at varied [Ca^2+^]o in diastole (minimum transient) and systole (maximum *F*/*F*_o_). BDL-cirrhotic trabeculae generate less minimum and maximum contractile force throughout the range of stimulation. (**B**): Time-to-peak Ca^2+^ transient (TTP) and time for Ca^2+^ transient to return to half maximum (THR). Data are the mean ± SEM of 51–58 myocytes per group. * *p* < 0.05 and *** *p* < 0.001 compared to the corresponding sham group. Calcium transients are decreased in cirrhotic trabeculae compared to controls (Reproduced from Honar H. et al. [16]).

We [18] studied cardiomyocytes isolated directly from both cirrhotic and sham-control rats to measure potassium currents and demonstrated that, compared with controls, two types of potassium currents are depressed in ventricular myocytes from cirrhotic rats, *I*(*t*), a calcium-independent transient outward K+ current, and *Isus*, a delayed-rectifier K+ current. These abnormalities may lengthen the action potential duration of cardiomyocytes from the cirrhotic heart. The prolonged action potential duration decreases the efficiency of SR calcium release and therefore prolongs cardiomyocyte contraction/relaxation. Sah et al. [42] found that early phase-1 repolarization of the action potential is critical for maintaining the optimal calcium spark and synchronization of release events; altered action potential induces asynchronous SR calcium release. We demonstrated that in BDL rats with cardiac dysfunction, i.e., cirrhotic cardiomyopathy, the kinetics of calcium rise slows. The prolongation of time-to-peak Ca^2+^ transient in our cirrhotic cardiomyocytes may be responsible for slowed contraction in BDL hearts. We further found that the maximum calcium transient is significantly smaller in cardiomyocytes from cirrhotic hearts compared with that from sham controls. Time-to-peak calcium transient in cardiomyocytes from cirrhotic hearts is significantly longer than that from sham controls (Figure 5). In addition to cardiac contractility, relaxation time also depends on intracellular calcium kinetics. Our research verified the slowed relaxation of cardiomyocytes from cirrhotic hearts.

Another factor that impacts cardiac contractility is the sarcomere length. Sato et al. [43] demonstrated that the longer the sarcomere is in a steady state, the more powerful the force. Tanner et al. [44] reported that the maximal calcium-activated force is increased in cardiomyocytes with longer sarcomeres; the calcium activity is also increased with the longer sarcomere. We showed that the sarcomere length is shorter at rest during full-relaxation in cirrhotic cardiomyocytes compared with sham controls, which may partially explain the decreased force-generating capacity of trabeculae from the cirrhotic heart compared with that from sham controls [16].

## 6. Abnormal Calcium Handling Decreases Cardiac Force-Generating Capacity

Shao et al. [45] evaluated the correlation between cardiomyocyte force-generating capacity and the impaired calcium transient in an experimental rat model of hypothyroid cardiomyopathy. They showed that the defects in the cardiomyocyte force-generating capacity and relaxation process result from impaired calcium regulation in hypothyroid cardiomyopathy. The mechanism is apparently up-regulated inducible NOS (iNOS) with a down-regulated β_1_-adrenergic receptor (β_1_-AR). We also demonstrated increased iNOS [46] and decreased β_1_-AR [47,48] in cirrhotic rat hearts.

Our lab [16] recently showed that force-generating capacity is significantly decreased in right ventricular trabeculae from BDL-cirrhotic rat hearts compared with sham controls (Figure 5) Moreover, this difference became more pronounced with increasing calcium concentration in the perfusion buffer. All the studies above indicate that the defects in calcium regulation are the essential driver of the altered cardiomyocyte force-generating capacity and relaxation in cirrhotic cardiomyopathy.

## 7. Cardiac Inflammation and Calcium Handling System

Our previous studies demonstrated the role of TNFα in cirrhotic cardiomyopathy [46,49]. We demonstrated that TNFα is increased in BDL-cirrhotic murine hearts, andnTNFα knockout or using an anti-TNFα antibody to neutralize TNFα significantly improved the depressed cardiac contractility in cardiomyocytes from the cirrhotic heart [49]. However, the exact mechanism remains unclear. Zuo and colleagues [50] added TNFα directly to atrial myocytes, and observed that TNFα directly increases spontaneous calcium release, decreases the amplitude of calcium transients, prolongs the decay times of calcium release, and reduces the SR calcium content. This study demonstrated that the acute effect of TNFα on the calcium handling system may explain the inhibitory impact of TNFα on contractile function in cirrhotic hearts. Because of the acute nature of the study, Zuo and coworkers could not clarify the long term role of TNFα, which is more relevant to the circumstances of cirrhotic cardiomyopathy.

Several studies demonstrated that TNFα modulates the expression of calcium regulatory proteins. Kao et al. [51] incubated cardiomyocytes with TNFα (50 ng/mL) for 24 h and showed that TNFα enhances methylation in the promoter region of SERCA2a. Furthermore, mRNA transcription and protein expression were significantly decreased. They concluded that inhibiting TNFα-induced hypermethylation may be a novel therapeutic strategy for cardiac dysfunction in heart failure. Rao et al. [52] investigated the effect of TNFα on the T-type calcium channel (TCC) which plays an important role in quicker depolarization, pressure-overload cardiac hypertrophy, and atrial fibrillation. They demonstrated that after culturing TNFα with atrial myocytes for 24 h, TCC is significantly reduced in a TNFα concentration-dependent manner. The peak calcium current was also reduced in a TNFα concentration-dependent manner. In addition to a structural change, the phosphorylation of the calcium handling protein also plays an important role in the genesis of cardiac dysfunction. Gregolin and coworkers [53] demonstrated that, although the quantities of intracellular proteins in the calcium handling system are not changed, inflammation significantly decreased the phosphorylation of phospholamban in thioacetamide-induced murine cirrhosis, which may explain the systolic and diastolic dysfunction. These findings provide molecular and functional insights into the effects of cirrhosis on cardiac function.

## 8. Oxidative Stress Disturbs Calcium Homeostasis

In rats with cirrhosis induced by bile duct ligation, oxidative stress is increased, which causes cardiac dysfunction [4,15]. However, the detailed pathogenic mechanism remains incompletely known. Oxidative stress has several impacts on calcium transients: (1) it impairs the function of the sodium–potassium exchanger, which causes calcium influx and sodium efflux. Our unpublished data indicated that the protein expression of the sodium–potassium exchanger is significantly decreased in cirrhotic hearts compared to that in sham controls; (2) it increases calcium influx through the L-type calcium channels; (3) it promotes ryanodine receptor 2 activity; and (4) it inhibits SR calcium-adenosine triphosphatase 2 (SERCA2) activity. All these effects cause calcium overload and reduce myofilament calcium sensitivity, eventually resulting in cardiac contractile dysfunction [54]. In addition to the effect of oxidative stress on the calcium handling system, it also affects other ions, such as potassium channels, sodium channels, and chloride channels [55]. These channels interact with each other and can impact contractile function.

## 9. Possible Therapeutic Strategies for Cirrhotic Cardiomyopathy

It is now clear that abnormalities of the calcium handling system play an essential role in cirrhotic cardiomyopathy. In addition to L-type calcium channels, RyR2 dispersion may also be a culprit in the pathogenesis of cardiac dysfunction in cirrhosis. Any agents that prevent RyR2 dispersion or restore RyR2 integrity of interdomain interactions may exert beneficial therapeutic effects on cirrhotic cardiomyopathy.

## 10. Antioxidants

Oxidative stress affecting RyR2 destabilizes interdomain interactions within the RyR2 (RyR2 dispersion) which causes calcium leakage from the SR and seems to play a key role in the pathogenesis of heart failure [56]. The antioxidant edaravone significantly stabilizes the interdomain interactions within the RyR2, restores the integrity of RyR2, reduces calcium leakage, and restores cardiac function to almost complete normalcy. The same group [57] later examined carvedilol, an alpha- and beta-adrenergic blocker, to treat canine pacing-induced heart failure, and observed that the interdomain interaction within RyR was defective in the failing heart, but the domain interaction remained normal in carvedilol-treated dogs, with preserved cardiac function. They further demonstrated that the beneficial properties of carvedilol were mediated via an antioxidative effect. Our study [15], demonstrated that oxidative stress plays a significant role in cirrhotic cardiomyopathy. Erythropoietin, an antioxidant, significantly decreased oxidative stress in the cirrhotic rat heart and reversed the impaired contractile function [15]. We speculate that the mechanism may be by restoring the integrity of RyR2.

## 11. β-Adrenergic Blockers

β-blockers are commonly used for the treatment of cardiac tachyarrhythmias, high blood pressure, and congestive heart failure. β-blockers are also administered to patients with cirrhosis, mainly for gastrointestinal bleeding [58]. Shen et al. demonstrated that prolonged β-adrenergic stimulation destabilizes the interdomain interactions of RyR2 in cardiomyocytes [28], which plays a key role in heart failure. β-blockers inhibit β-adrenergic stimulation and therefore prevent RyR2 dispersion, restoring the interdomain interactions to normal. Martinez-Hernandez and Blatter [59] studied isolated rabbit atrial myocytes to test the effect of carvedilol on SR calcium release. They observed that carvedilol prevents spontaneous calcium release from cellular SR and significantly reduces spontaneous calcium waves. Therefore, β-blockers exert beneficial therapeutic effects food on cardiac tachyarrhythmias and heart failure via modulation of RyR2.

## 12. Bile Acids

Another possible therapeutic strategy is targeting bile salts. Desai et al. [60] analyzed 40 children with biliary atresia and reported that 52% had abnormal cardiac morphologies such as increased left ventricular mass, left atrial volume index, and left ventricular internal diameter. Thus, these patients manifested pediatric CCM. In another study, this group used 3,5-diethoxycarbonyl-1,4-dihydroxychollidine (DDC) to create a biliary fibrosis model in mice. The authors demonstrated that DDC causes cardiac structural, functional, electrocardiographic, and molecular abnormalities and reduces cardiac response to catecholamines. The cardiac changes were reversed following the resolution of the biliary fibrosis. Bile acids are thus a key mediator of cardiac dysfunction, and decreasing bile acid levels or changing the bile acid pattern may be an effective treatment of cirrhotic cardiomyopathy [61].

In conclusion, the calcium handling system plays a key role in non-cirrhotic and cirrhotic cardiovascular diseases. Many etiologic factors that play important roles in cirrhotic cardiomyopathy also impact the calcium handling system, such as TNFα and oxidative stress. The factors that inhibit cardiac contractility in cirrhotic cardiomyopathy may act by impacting the cardiomyocyte calcium handling system.

## Figures and Tables

**Table 1:** Prevalence of CCM according to the two diagnostic criteria (modified from Chahal et al. [4]).

Authors	2005 WGC	2019 CCC	*p*-Value
Razpotnik et al. [5]	66.2%	55.7% with GLS < 18% and >22% 19.7% with GLS < 18% only	*p* = NS *p* < 0.05
Singh et al. [6]	74.8%	85.6% with GLS < 18% only	*p* = NS
Spann et al. [7]	77%	30% without GLS	*p* < 0.05
Ali et al. [8]			
Cesari et al. [9]		29% including GLS < 18% and >22%	
Izzy et al. [10]		38.8%	

WCG: World Congress of Gastroenterology, CCC: Cirrhotic Cardiomyopathy Consortium, and GLS: global longitudinal strain. NS: non-significant.

## Data Availability

Not applicable.
